# Indirect reduction of *Ralstonia solanacearum* via pathogen helper inhibition

**DOI:** 10.1038/s41396-021-01126-2

**Published:** 2021-10-20

**Authors:** Mei Li, Thomas Pommier, Yue Yin, Jianing Wang, Shaohua Gu, Alexandre Jousset, Joost Keuskamp, Honggui Wang, Zhong Wei, Yangchun Xu, Qirong Shen, George A. Kowalchuk

**Affiliations:** 1 Jiangsu Provincial Key Lab for Organic Solid Waste Utilization, Key Lab of Plant Immunity, Jiangsu Collaborative Innovation Center for Solid Organic Waste Resource Utilization, National Engineering Research Center for Organic-based Fertilizers, Nanjing Agricultural University, 210095 Nanjing, PR China; 2 Institute for Environmental Biology, Ecology & Biodiversity, Utrecht University, Padualaan 8, 3584CH Utrecht, The Netherlands; 3 Univ Lyon, Université Claude Bernard Lyon 1, CNRS, INRAE, VetAgro Sup, UMR Ecologie Microbienne, F-69622 Villeurbanne, France; 4 Center for Quantitative Biology and Peking-Tsinghua Center for Life Sciences, Peking University, Beijing, PR China; 5 Biont Research, Abeelstraat 33, 3552 RC Utrecht, The Netherlands; 6 School of Environmental Science and Engineering, Yangzhou University, 225127 Yangzhou, Jiangsu PR China

**Keywords:** Microbial ecology, Microbial ecology, Microbial ecology

## Abstract

The rhizosphere microbiome forms a first line of defense against soilborne pathogens. To date, most microbiome enhancement strategies have relied on bioaugmentation with antagonistic microorganisms that directly inhibit pathogens. Previous studies have shown that some root-associated bacteria are able to facilitate pathogen growth. We therefore hypothesized that inhibiting such pathogen helpers may help reduce pathogen densities. We examined tripartite interactions between a model pathogen, *Ralstonia solanacearum*, two model helper strains and a collection of 46 bacterial isolates recovered from the tomato rhizosphere. This system allowed us to examine the importance of direct (effects of rhizobacteria on pathogen growth) and indirect (effects of rhizobacteria on helper growth) pathways affecting pathogen growth. We found that the interaction between rhizosphere isolates and the helper strains was the major determinant of pathogen suppression both in vitro and in vivo. We therefore propose that controlling microbiome composition to prevent the growth of pathogen helpers may become part of sustainable strategies for pathogen control.

## Introduction

Plant pathogens have a large negative impact on agricultural production, and there is an urgent need for sustainable strategies to prevent diseases while reducing the environmental footprint of modern agriculture [1]. Plant root-associated microorganisms are increasingly studied in relation to their ability to help keep plants healthy [2, 3]. However, while some microbiomes are better at preventing pathogen growth than others, it often remains unclear which interactions shape pathogen success. To date, most research has focused on pathogen inhibition by some specific plant-associated microorganisms. In line with this logic, most microbiome management strategies have been centered around bioaugmentation with microorganisms that can directly inhibit pathogen growth [4, 5]. These biopesticides represent a promising approach, but are often constrained by the low density that inoculated strains can reach in a multispecies microbiome and the context-dependent success of microbial introductions [6–8]. These shortcomings are at least partly due to inadequate consideration of the complex microbial interactions that impact pathogen inhibition or proliferation [9, 10].

We propose a new perspective in pathogen ecology by placing focus on microorganisms that promote pathogen growth. Recent studies have shown that a significant fraction of plant-associated microorganisms can promote pathogen growth and pathogenicity [11]. Facilitative microbe-microbe interactions are indeed widespread, and such interactions may emerge for instance as a result of cross-feeding [12] or production of public goods such as siderophores [13]. Facilitation has been recently highlighted as a potential determinant of pathogen success [9, 14]. We therefore postulate that affecting naturally-occurring helper bacteria of pathogens may provide an alternative means of controlling pathogen development, as compared to the application of pesticides or biopesticides. To this end, we hypothesized that indirect effects via inhibition of pathogen helpers would have a significant impact on realized pathogen densities and subsequent disease incidence.

**Fig. 1:**
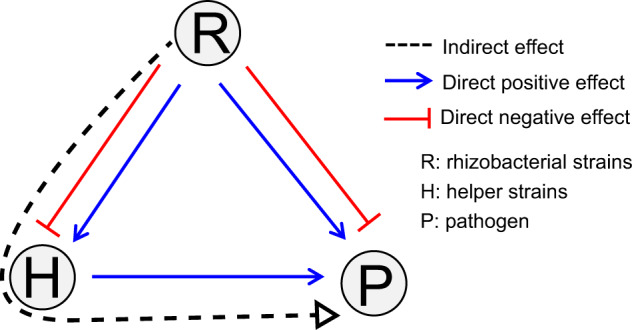
Conceptual overview of direct and indirect effects of rhizobacterial strains on pathogen growth. In this work, we subdivided the net, apparent effect of single rhizosphere bacterial isolates (R) on pathogen density into direct effects on the pathogen (P) and indirect effects mediated by interactions with helper bacteria (H).

We first established the prevalence of pathogen-helper bacteria in the rhizosphere by screening a library of 640 rhizobacterial strains isolated from tomato rhizosphere soil. We specifically tested their pairwise interactions with *Ralstonia solanacearum*, the causative agent of bacterial wilt, a major disease affecting more than 200 crops at a global scale [15, 16]. We observed that a significant fraction of all isolates promoted pathogen growth in vitro. We then selected two representative pathogen helper strains and built tripartite cultivation experiments in which the pathogen was grown together with one of the helper strains and the supernatants of 46 individual bacterial strains chosen to represent a gradient of positive, neutral or negative interactions with the pathogen. Pathogen growth was monitored in each community, both in vitro and in the tomato rhizosphere. We then expressed pathogen density and disease severity as a function of a direct (effect on pathogen) and indirect (effect on the helper strain) pathways for each of these rhizobacteria (Fig. 1). Part of the resulting data was also used to model the relative importance of direct versus indirect effects in determining realized pathogen density and subsequent disease severity. Based upon the results of these experiments, we discuss the potential utility of bioaugmentation strategies that target pathogen helpers as an element of integrated pathogen control.

## Materials and methods

### Rhizosphere soil sampling

A total of 20 rhizosphere soil samples (20 tomato plants) were collected at the flowering stage from a tomato field located in Qilin town, Jiangsu province, China, 118°57’ E, 32°03’ N, which had been infested by the pathogen *Ralstonia solanacearum* for more than 15 years [8]. After uprooting plants, excess soil was first gently shaken from the roots, and the remaining soil attached to roots was considered as rhizosphere soil. Each rhizosphere soil sample was then used for bacterial strain isolation.

### Isolation and identification of rhizobacteria

#### Isolation

A total of 640 bacterial strains were isolated from the fresh rhizosphere soil samples, according to a previously established protocol [11]. Briefly, 1 g of each rhizosphere sample was mixed with 9 mL MS buffer solution (50 mM Tris-HCl [pH 7.5], 100 mM NaCl, 10 mM MgSO_4_, 0.01% gelatin) in a rotary shaker at 170 rpm min^−1^ for 30 min at 30 °C. After serial dilution in MS buffer solution, 100-μl volumes of the diluted soil suspensions were plated on 1/10 tryptone soy agar (1/10 TSA, 1.5 g L^−1^ tryptone, 0.5 g L^−1^ soytone, 0.5 g L^−1^ sodium chloride, and 15 g L^−1^ agar, pH 7.0). After a 48-h incubation at 30 °C in the dark, 32 isolates were randomly picked per rhizosphere soil sample. To avoid potential fungal contamination, only highly diluted samples were used for isolation. The isolates were then re-streaked on TSA plates for colony purification. Approximately 5.5% (35 isolates) of the bacterial isolates failed to grow on the TSA plates for unknown reasons when we re-streaked them and were therefore omitted from the dataset. The final collection thus consisted of 605 bacterial isolates derived from 20 rhizosphere soil samples. All purified isolates were cultured in 100 μl tryptone soy broth (TSB, liquid TSA) in 96-well microtiter plates at 30 °C with shaking (rotary shaker at 170 rpm) for 18 h before freezing and storing at −80 °C in 15% glycerol.

#### Strain identification

We sequenced the full 16 S rRNA gene to taxonomically identify all 605 rhizobacterial isolates. The 16 S rRNA gene was sequenced via Sanger sequencing of PCR products from glycerol stocks by Shaihai Songon Biotechnology Co., Ltd, Shaihai Station. The PCR system (25 µl) was composed of 1 µl of bacterial cells (overnight culture), 12.5 µl mixture, 1 µl of forward (27 F: 5-AGA GTT TGA TCA TGG CTC AG-3) and reverse primer (1492 R: 5-TAC GGT TAC CTT GTT ACG ACT T-3) each [17] and 9.5 µl of sterilized water. PCR was performed by initially denaturizing at 95 °C for 5 min, cycling 30 times with a 30-s denaturizing step at 94 °C, annealing at 58 °C for 30 s, extension at 72 °C for 1 min 30 s, and a final extension at 72 °C for 10 min. The 16 S rRNA gene sequences were identified using NCBI databases and homologous sequence similarity. A total of 90 bacterial isolates that were identified as *Ralstonia solanacearum* were removed from further analyses, resulting in 515 remaining isolates.

### Direct effect of rhizobacteria on pathogen growth in vitro

We used *R. solanacearum* strain QL-Rs1115 tagged with the pYC12-mCherry plasmid as a model bacterial pathogen [8, 18]. We first tested the direct effects of the 515 non-*R. solanacearum* bacterial strains on the growth of *R. solanacearum* in vitro by using supernatant assays. Briefly, after 48 h of growth in NB (nutrient broth) medium (glucose 10.0 g l^−1^, tryptone 5.0 g l^−1^, yeast extract 0.5 g l^−1^, beef extract 3.0 g l^−1^, pH 7.0) on a shaker at 170 rpm, 30 °C, all bacterial cultures were filter sterilized to remove living cells (0.22 µm filter). Subsequently, 20 µl of sterile supernatant from each strain’s culture and 2 µl overnight culture of the pathogen (adjusted to OD600 = 0.5 after 12 h growth at 30 °C with shaking) were added into 180 µl of fresh NB medium (5-times diluted, in order to better reflect the effect of the supernatant). Control treatments were inoculated with 20 µl of 5 X diluted NB media instead of the bacterial supernatant. Each treatment was conducted in triplicate. All bacterial cultures were grown for 48 h at 30 °C with shaking (170 rpm) before measuring pathogen density as red mCherry protein fluorescence intensity (excitation: 587 nm, emission: 610 nm) [9, 11] which was linearly related to the CFU of pathogen *R. solanacearum* (Fig. S1). To test for significance of growth promotion or inhibition, *R. solanacearum* densities were log_10_-transformed prior to analyses of variance (ANOVA) and Bonferroni *t* test to compare mean differences between each rhizobacterial supernatant treatment and the control treatment, with *p* values less than 0.05 considered statistically significant. The effect on pathogen growth was defined as the percentage of improvement or reduction in pathogen growth by the supernatant compared to the control treatment. When the effect on pathogen growth was positive, i.e., when the supernatants from strains significantly promoted the growth of the pathogen, they were considered as helpers of the pathogen. If the effect on pathogen growth was negative, i.e., when the supernatants from strains significantly inhibited the growth of the pathogen, they were considered as inhibitors of the pathogen.

### Assessing strain redundancy among the 515 non-*Ralstonia solanacearum* bacteria

We assessed possible redundancy among the 515 strains of the non-*Ralstonia solanacearum* rhizobacteria. To encompass both taxonomic and functional redundancies, we considered the 16 S rRNA gene sequences as well as the direct effect of their supernatant on *Ralstonia solanacearum*. Self BLAST searches were performed on the full 515 sequence dataset using the *makeblastdb* and *blastn* commands from the BLAST command line tool [19]. Sequences showing >99% identity over >95% of the full length of the 16 S rRNA gene were considered as taxonomically redundant. We then compared the direct effects on pathogen growth of the taxonomically redundant strains, and removed those showing the same patterns of interactions (positive, negative or neutral). Accordingly, (see the dataset “Library of rhizobacterial strains” in the supplementary information), 355 of the 515 strains (68.9%) were removed from the original dataset for further analyses.

### Phylogenetic tree construction

**Fig. 2:**
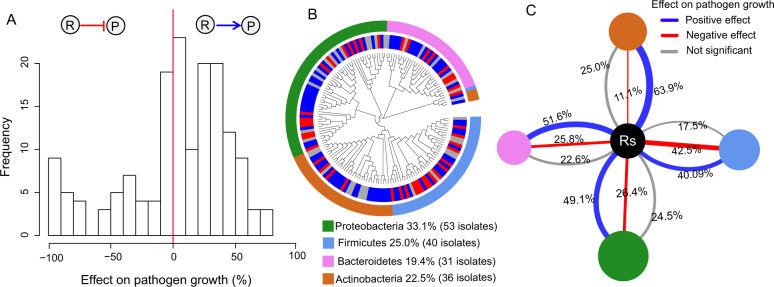
Taxonomic characterization of rhizobacterial isolates that inhibited or helped the growth of *Ralstonia solanacearum*. **A** Distribution of in vitro effects of 160 rhizobacterial supernatants on *R. solanacearum* growth. The red vertical line represents no effect on *R. solanacearum* growth. **B** Cladogram depicting the phylogenetic relationship among the 160 isolates based on their full-length 16 S rRNA gene sequences. The inner ring depicts the different effect of isolates supernatant on *R. solanacearum* growth: positive effect (blue), negative effect (red) and no significant effect (gray). The outer ring shows the four phyla to which the isolates belong. **C** The proportion of rhizobacterial isolates per phylum whose supernatant showed inhibitory, stimulatory or no effect on *R. solanacearum* growth. The size of the circles represents the number of rhizobacterial isolates in the given phylum. The thickness of lines represents the percentage of rhizobacterial isolates that have the indicated effect on *R. solanacearum* growth in each phylum.

The 16 S rRNA gene sequences of the 160 non*-*redundant bacteria were aligned using MUSCLE [20]. Sequences in the alignment were trimmed at both ends to obtain maximum overlap using the MEGA X software, which was also used to construct taxonomic cladograms [21]. We constructed a maximum-likelihood (ML) tree, using a General Time Reversible (GTR) + G + I model, which yielded the best fit to our data set. Bootstrapping was carried out with 100 replicates retaining gaps. A taxonomic cladogram was created using the EVOLVIEW web tool (https://evolgenius.info//evolview-v2/). To show the relationship between phylogeny and the effects of rhizobacteria on pathogen growth, we added taxonomic status (phylum) of each rhizobacterial strain and its effect on pathogen growth as heatmap rings to the outer circle of the tree separately (Fig. 2B).

### Effects of rhizobacteria on pathogen helper strains growth in vitro

**Fig. 3:**
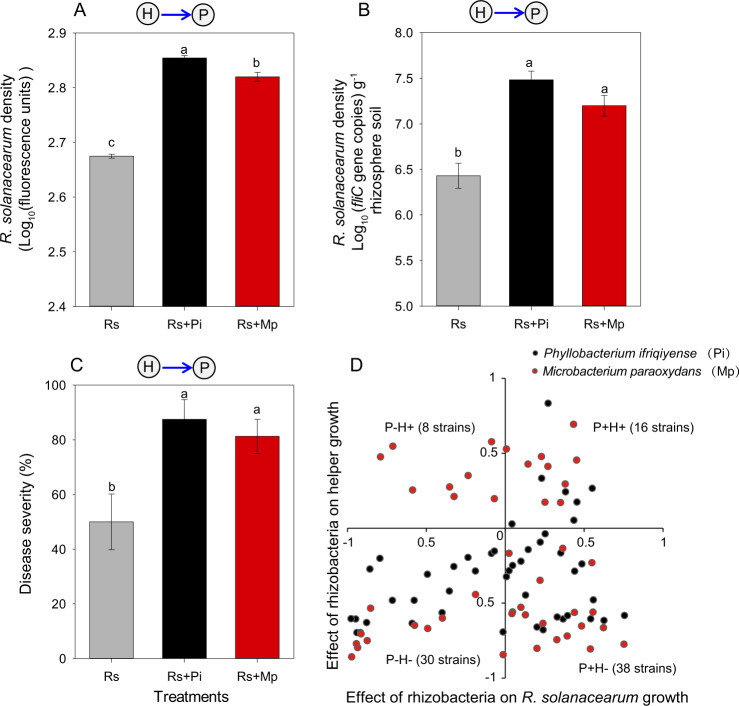
Effect of helper strains on *Ralstonia solanacearum* growth and plant disease severity. Effects of the two helper strains *Phyllobacterium ifriqiyense* (Pi) and *Microbacterium paraoxydans* (Mp) on *Ralstonia solanacearum* (Rs) growth in vitro (**A**) and in vivo (**B**) and on plant disease severity (**C**). Different letters indicate significant differences based on Tukey post hoc test. Error bars show ±1 SE (*n* = 3 for in vitro, *n* = 4 for in vivo). **D** Effects of 46 rhizobacterial strains on the growth of *R. solanacearum* and the two model helper strains in vitro. The x-axis shows the direct effect of each rhizobacterial strain on *R. solanacearum* growth (data from the experiment in which *R. solanacearum* was grown in the presence of supernatant from each of the 46 rhizobacterial strains—the same data is presented on the x axis of Fig. 4A). The y-axis shows the effect of each rhizobacterial strain on each of the two helper strains (data from the experiment in which each helper was grown in the presence of supernatant from each of the 46 rhizobacterial strains—the same data is presented on the x axis of Fig. 4C). In (**C**), “−1”, “0” and “1” on the x-axis denote that *R. solanacearum* growth is completely inhibited, not influenced or increased 2× by supernatant from the rhizobacteria, respectively. Similarly, “−1”, “0” and “1” on the y-axis denote the same growth effects with reference to growth of the helper strains. Black dots indicate results involving interactions with Pi, and red dots indicate results involving interactions with Mp.

**Fig. 4:**
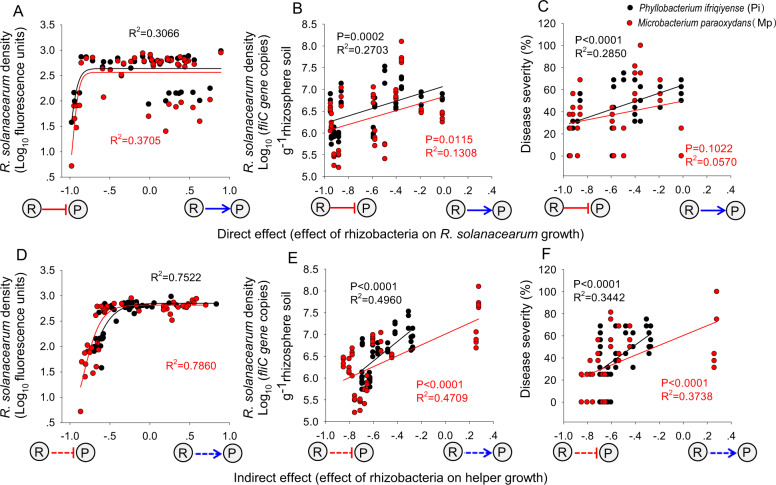
The importance of direct versus indirect effects on *Ralstonia solanacearum* density and disease severity in the presence of helper strains. In the presence of helper *Phyllobacterium ifriqiyense* (Pi) or *Microbacterium paraoxydans* (Mp), respectively, the importance of direct effects on the density of *R. solanacearum* both (**A**) in vitro and (**B**) in vivo, and (**C**) disease severity (the data on the x axis of (**A**) are the same data which was presented on the x axis of Fig. 3C, the data on x axis of (**B**) and (**C**) are part of the data on x axis of (**A**)); the importance of indirect effects on the density of *R. solanacearum* both (**D**) in vitro and (**E**) in vivo, and (**F**) disease severity (the data on the x axis of (**D**) are the same data which was presented on the y axis of Fig. 3C, the data on x axis of (**E**) and (**F**) are part of the data on x axis of (**D**)). In all panels, “−1”, “0” and “1” on the x-axis denote that *R. solanacearum* growth (**A**, **B**, and **C**) or helper growth (**D**, **E**, and **F**) is completely inhibited, not influenced or increased 2× by supernatant from the rhizobacteria, respectively.

We then assessed the potential of different rhizosphere isolates to inhibit helper strains. We first selected two model helper strains (*Phyllobacterium ifriqiyense LM1* (Pi) and *Microbacterium paraoxydans LM2* (Mp)), which showed strong positive effects on pathogen growth both in co-culture and in supernatant assays (Fig. S2). We defined the effect of rhizobacterial strains on the growth of helpers as the indirect effect on *R. solanacearum* growth. To study these indirect effects, we first chose a subset of 46 rhizobacterial strains representing a gradient of positive, neutral or negative effect on pathogen growth based on supernatant assays (results in x axis of Figs. 3C and 4A, B, C). We then tested the effects of these 46 rhizobacterial strains on the growth of each of the two helper strains using supernatant assays. Briefly, after 48 h growth in NB media, each of the 46 bacterial monocultures was passed through a 0.22 µm filter to remove living cells. Then 20 µl of sterile supernatant from each strain’s culture and 2 µl overnight culture of Pi or Mp (adjusted to OD_600_ = 0.5 after 12 h growth at 30 °C with shaking) were added into 180 µl of fresh NB medium (5-times diluted, in order to better reflect the effect of the supernatant). Control treatments were inoculated with 20 µl of 5× diluted NB media instead of a bacterial supernatant. Each treatment was replicated four times. All bacterial cultures were grown for 24 h at 30 °C with shaking (170 rpm) before measuring helper density as optical density (OD_600_). To test for significance of growth promotion or inhibition, we used analyses of variance (ANOVA) and Bonferroni *t* test to compare mean differences of helper density between each rhizobacterial supernatant treatment and the control treatment, with *p* values lower than 0.05 being considered statistically significant. The effect of rhizobacteria on the helpers’ growth (results in y axis of Fig. 3C and x axis of Fig. 4D, E, F) was defined as the percentage of increase or reduction in helper growth by the supernatant compared to the control treatment.

### In vitro pathogen growth in the presence of a helper strain and supernatant from rhizobacterial isolates

To disentangle the direct effects from the indirect effects of rhizobacteria on *R. solanacearum* growth, we compared their relative effects using in vitro triculture assays comprised of *R. solanacearum*, one of the two helper strains and supernatant of one of the 46 chosen rhizobacterial strains. Briefly, after 48 h of growth in NB media, each of the 46 bacterial monocultures was passed through a 0.22 µm filter to remove living cells. Then, 20 µl of sterile supernatant from each strain’s culture and 2 µl overnight culture of Pi or Mp (densities were adjusted to ~10^7^ cells per ml) were added to 180 µl of fresh NB medium (5-times diluted). Each treatment was replicated four times. At the same time, 2 µl overnight culture of mCherry-tagged *R. solanacearum* (density was adjusted to ~10^6^ cells per ml) was added to each treatment in 96-well plates at 30 °C with shaking (170 rpm). After 24-h growth, *R. solanacearum* density (results in y axis of Fig. 4A, D) was measured as the red mCherry protein fluorescence intensity (excitation: 587 nm, emission: 610 nm) with a SpectraMax M5 plate reader.

### In vivo pathogen growth and plant disease development in the presence of a helper strain and a rhizobacterial strain

To validate in vitro results, we set up greenhouse experiments where plants were inoculated with a bacterial consortium consisting of *R. solanacearum*, one of the two helper strains and a test rhizobacterial strain. Tomato seeds (*Lycopersicon esculentum*, cultivar “*Ai hong sheng*”) were surface-sterilized by soaking them in 3% NaClO for 5 min and in 70% ethyl alcohol for 1 min before being germinated on water-agar plates for 2 days. Seeds were then sown into seedling trays containing gamma irradiation-sterilized (to avoid potential effects of the resident community) seedling substrate (Huainong, Huaian Soil and Fertilizer Institute). At the three-leaf stage, tomato plants were transplanted to seedling trays containing 200 g of the same seedling substrate as describe above.

To relate our results to practical application conditions, we selected a subset of 12 strains that displayed a range of inhibitions effects on pathogen and helpers (Table S1) out of the 46 rhizobacterial isolates used for the in vitro assays. Each rhizobacterial strain was used in combination with each of the two helper strains and *R. solanacearum*, resulting in a total of 28 treatments (Table S2), including a water control, *R. solanacearum* alone, and *R. solanacearum* with just each of the two helper strains (results in Fig. 3B, C). For each treatment, four replicate seedling trays were used, with each replicate seedling tray containing 4 tomato plants. Three days after transplantation, plants of each treatment were inoculated with one of the two helper strains, alone or in combination with one of the rhizobacterial strains, using the root drenching method at a final concentration of 10^8^ CFU g^−1^ soil for each bacterial strain [22]. Seven days after inoculation of helper alone or together with rhizobacteria, *R. solanacearum* was introduced to the roots of all plants at a final concentration of 10^7^ CFU g^−1^ soil. The positive control treatment with *R. solanacearum* alone was inoculated only with the pathogen, and the negative control treatment was not inoculated with any bacteria. Tomato plants were maintained under standard greenhouse conditions (i.e., at natural temperature variation ranging from 28 °C to 32 °C, 15/9 h day/night conditions) and watered regularly with sterile water. Seedling trays were rearranged randomly every two days. Forty days after transplantation, plants were destructively harvested. The disease index for each plant was recorded based on a scale ranging from 0 to 4 [23]. Disease severity for each replicate seedling plate was calculated as described by: Disease severity = [∑ (The number of diseased plants in the disease index category × disease index category)/ (Total number of plants used in the experiment × highest disease index category)] ×100% [23, 24]. Simultaneously, we collected rhizosphere soil samples following an established protocol [4]. Briefly, two plants were randomly chosen from each replicate seedling tray to collect rhizosphere soils and further combined to yield one sample, resulting in a total of 112 rhizosphere soil samples for which *R. solanacearum* population densities were determined.

### Quantification of *R. solanacearum* at the end of the in vivo experiment

We determined *R. solanacearum* densities using quantitative PCR (qPCR). DNA was extracted from rhizosphere soils using a Power Soil DNA isolation kit (Mo Bio Laboratories) following the manufacturer’s protocol. DNA concentrations were determined by using a NanoDrop 1000 spectrophotometer (Thermo Scientific) and extracted DNA was used for *R. solanacearum* density measurements using specific primers (forward, 5ʹ-GAA CGC CAA CGG TGC GAA CT-3ʹ; reverse, 5ʹ-GGC GGC CTT CAG GGA GGT C-3ʹ) targeting the *fliC* gene, which encodes the *R. solanacearum* flagellum subunit [25]. The qPCR analyses were carried out with a StepOnePlus Real-Time RCR Instrument using SYBR green fluorescent dye detection and three technical replicates as described previously [4].

### Statistical analyses

To meet assumptions of normality and homogeneity of variance, *R. solanacearum* densities measured in vitro and in vivo were log10-transformed. When comparing mean differences between treatments, we used analyses of variance (ANOVA) and the Tukey Test, where *p* values lower than 0.05 were considered statistically significant. *R. solanacearum* densities were explained by two quantitative indices, the direct effect of rhizobacteria on *R. solanacearum* growth (the effect of rhizobacteria on *R. solanacearum* growth) and the indirect effect of rhizobacteria on *R. solanacearum* growth (the effect of rhizobacteria on helper strains’ growth). Nonlinear regression analyses (Sigmoidal, Sigmoid, 3 Parameter) were used to analyze the relationship between the direct effect and pathogen density, as well as the relationship between indirect effects and pathogen density in the presence of helper strains in vitro. The relationships between them, and between direct/indirect effects and disease severity in the presence of helper strains in vivo, were analyzed using linear regressions. These analyses were carried out using the R 3.6.3 program (www.r-project.org) and Sigma Plot (V.12.5).

**Fig. 5:**
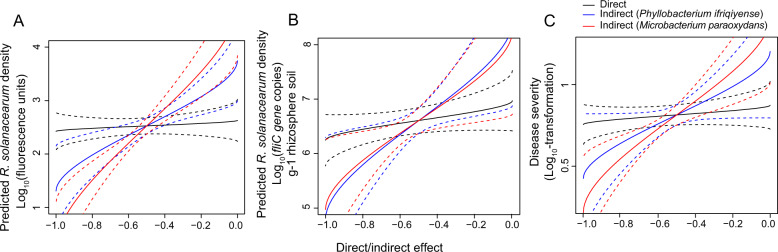
The relative importance of direct versus indirect effects on *Ralstonia solanacearum* density and disease severity in the presence of helper strains. Relative importance of direct versus indirect effects on *Ralstonia solanacearum* density both in vitro (**A**) and in vivo (**B**), and disease severity (**C**) in presence of helper strains on the interaction scenario where rhizobacterial strains inhibited both the pathogen and its helpers (quadrant “H^−^P^−^” in Fig. 3C). This shows the predicted *R. solanacearum* densities and disease incidence for different values of the inhibition via pathogen (Direct) or helper (Indirect) as estimated from the statistical model (Table 1) which with direct effects, indirect effects, and an interaction between helper strains and indirect effects as fixed factors. For the Direct line, the indirect effect was set to zero, while for the indirect line, the direct effect was set to zero.

To further consider the growth inhibition of *R. solanacearum*, and disease suppression, we fitted a linear model to estimate the relative importance of direct effects versus indirect effects on the density of *R. solanacearum* both in vitro and in vivo, and on disease severity. This model considered the interaction scenario where rhizobacterial strains inhibited both the pathogen and its helpers (see the R script “Model” in the supplementary information). These analyses were performed in R version 3.6.3 [26] in conjunction with the package *car, readxl* and *dplyr*, and *tidyverse* 1.2.1 [27]. Briefly, proportional effects were normalized using a folded cube root transformation as suggested in J.W. Tukey [28] and fitted using a linear model with direct effects, indirect effects, and an interaction between helper strains and indirect effects as fixed factors. Normality of residuals was tested using the Shapiro-Wilk normality test and visual inspection of QQ-plots with standardized residuals. Type-II sum of squares were calculated using the ANOVA function from *car* 3.0-2 [29]. Subsequent visualization of the model outcome (results in Fig. 5) showed the predicted *R. solanacearum* densities and disease severity for different values of the inhibition via pathogen (Direct) or helper (Indirect) as estimated from the statistical model. For the Direct effect line, the indirect effect is set to be zero, while for the Indirect effect line, the direct effect is set to be zero.

## Results

### Taxonomic characterization of inhibiting and helping strains of *Ralstonia solanacearum* from the tomato rhizosphere

The 160 non-redundant isolates we examined were classified within four main phyla, with the following distribution: *Proteobacteria* 33.1%, *Firmicutes* 25.0%, *Bacteroidetes* 19.4% and *Actinobacteria* 22.5%. This collection contained a total of 23 families and 48 genera (Fig. S3). A total of 26.9% of these isolated rhizobacteria were shown to inhibit pathogen growth in vitro, while 50.6% of them significantly stimulated pathogen growth. We refer to these two categories as pathogen inhibitors and helpers, respectively (Fig. 2A). Although both helpers and inhibitors were found within each represented phylum, there were clear phylum-level differences with respect to the relative proportion of inhibitors versus helpers (Fig. 2B). For instance, 42.5% of the isolates affiliated with the *Firmicutes* showed inhibition of *R. solanacearum* growth, while 49.1% of the *Proteobacteria* isolates, 51.6% of the *Bacteroidetes* isolates and 63.9% of the *Actinobacteria* isolates were found to act as helpers (Fig. 2C).

### Pairwise interactions between helper strains of *R. solanacearum* and other rhizobacterial strains

To examine direct versus indirect effects on pathogen growth, we first chose two model helper strains: *Phyllobacterium ifriqiyense LM1* (Pi) and *Microbacterium paraoxydans LM2* (Mp). The helper strain Pi increased *R. solanacearum* density by 51.2% in vitro (Fig. 3A), by 946.7% (from 10^6.4^ to 10^7.4^) when grown in vivo with tomato plants (Fig. 3B), and increased disease severity by 75% (Fig. 3C). Similarly, Mp increased *R. solanacearum* density by 39.7% in vitro (Fig. 3A), by 461.6% (from 10^6.43^ to 10^7.20^) in vivo (Fig. 3B), and increased disease severity by 62.5% (Fig. 3C).

We then selected 46 rhizobacterial strains from the full rhizobacterial strain collection to represent a range of positive (50.0%), negative (34.8%) or neutral (15.2%) effects on *R. solanacearum* growth (Fig. S4). We defined these interactions as the *direct effect* of rhizobacteria on *R. solanacearum* growth (x axis of Figs. 3C and  4A, B, C). We tested the effects of the supernatant from each of these 46 rhizobacterial strains on each of the helper strains, Mp and Pi. We found that 10.9% of the strains positively affected the growth of Pi, while 82.6% reduced Pi growth and 6.5% had no significant effect (Fig. S4). Following a distinct but comparable pattern, Mp was positively affected by 37.0% of the tested isolates and negatively by 63.0% of them (Fig. S4). We defined these interactions as the *indirect effects* of rhizobacteria on *R. solanacearum* growth (y axis of Fig. 3C and x axis of Fig. 4D, E, F).

When considering the direct effect of each rhizobacterial strain with their indirect effects on the growth of *R. solanacearum*, four possible combinations were considered (Fig. 3C): (i) 8 strain combinations showed negative direct effects and positive indirect effects (P^−^H^+^), (ii) 16 strain combinations showed positive direct effects and positive indirect effects (P^+^H^+^), (iii) 30 strain combinations showed negative direct effects and negative indirect effects (P^−^H^−^) and (iv) 38 strain combinations showed positive direct effects and negative indirect effects (P^+^H^−^). A large majority of strain combinations fell into two of these categories, with 32.6% being P^−^H^−^ and 41.3% being P^+^H^−^, suggesting that indirect negative effects may be relevant to reducing the growth of *R. solanacearum*. Given this distribution and desire to examine pathways toward *R. solanacearum* inhibition, we focused subsequent modeling work (described in Fig. 5 and Table 1) on the “P^−^H^−^” quadrant, to examine the relative importance of direct effects versus indirect effects on the density of *R. solanacearum* and plant disease severity.

**Table 1:** ANOVA table comparing the contribution of direct and indirect effects of the different tested bacterial isolates on the density of *Ralstonia solanacearum* in vitro and in vivo, as well as disease severity on the interaction scenario where rhizobacteria inhibited both the pathogen and its helpers (quadrant “H^-^P^-^” in Fig. 3C).

	*R. solanacearum* density in vitro			*R. solanacearum* density in vivo			Disease severity		
	*df*	*F*	*P*	*df*	*F*	*P*	*df*	*F*	*P*
Direct effect	1	2.4521	0.1295	1	2.7504	0.1146	1	1.0440	0.3204
Indirect effect	1	32.9556	4.818e–06	1	11.5760	0.0032	1	14.3290	0.0014
Indirect effect: Mp vs Pi	1	5.0717	0.0330	1	0.0376	0.8485	1	0.7262	0.4053
No. of residuals	26			18			18		

### The importance of direct versus indirect effects on *R. solanacearum* density and plant disease severity in the presence of helper strains

In the presence of the helper strain Pi, the direct effects of the rhizobacteria explained a significant proportion of the variation in *R. solanacearum* density in vitro (*R*^2^ = 0.3066, black line in Fig. 4A) and in vivo (*R*^2^ = 0.2703, *P* = 0.0002, black line in Fig. 4B), as well as the level of bacterial wilt disease severity observed (*R*^2^ = 0.2850, *P* < 0.0001, black line in Fig. 4C). The indirect effects of the rhizobacteria explained a larger proportion of the observed variation in *R. solanacearum* density as compared to the direct effects for the in vitro assay (*R*^2^ = 0.7522, black line in Fig. 4D) and the in vivo assay (*R*^2^ = 0.4960, *P* < 0.0001, black line in Fig. 4E), as well as for the observed level of bacterial wilt disease incidence (*R*^2^ = 0.3442, *P* < 0.0001, black line in Fig. 4F).

When in the presence of the helper strain Mp, the direct effects on *R. solanacearum* density were again significant both in vitro (*R*^2^ = 0.3705, red line in Fig. 4A) and in vivo (*R*^2^ = 0.1308, *P* = 0.0115, red line in Fig. 4B), but the direct effects did not correlate significantly with bacterial wilt disease severity (red line in Fig. 4C). In the presence of this helper strain, *R. solanacearum* density was again correlated with indirect effects in vitro (*R*^2^ = 0.7860, red line in Fig. 4D) and in vivo (*R*^2^ = 0.4709, *P* < 0.0001, red line in Fig. 4E), as well as with the level of bacterial wilt disease severity (*R*^2^ = 0.3738, *P* < 0.0001, red line in Fig. 4F).

In the presence of either helper, Pi or Mp, the indirect effects explained more of the total variation in *R. solanacearum* density and disease severity than the direct effects, with the regression for indirect effects yielding higher r-square values than that for direct effects (Fig. 4A–F). Together, these results demonstrate that inhibition of pathogen helper strains has the potential to limit the growth of *R. solanacearum* both in vitro and in vivo, and to reduce of bacterial wilt disease severity. To gain further insight into the potential prevalence of such a mechanism, we considered this strategy using a modeling approach targeting the relative importance of direct versus indirect effects on pathogen growth and disease severity.

### Relative contribution of direct versus indirect effects on *R. solanacearum* density and disease severity in the presence of helper strains

To further consider growth inhibition of *R. solanacearum* and decrease in bacterial wilt disease severity, we focused our modeling approach on the interaction scenarios where rhizobacterial strains inhibited both the pathogen and its helpers (quadrant “H^−^P^−^” in Fig. 3C). We constructed a model to predict the direct effects versus indirect effects on the density of *R. solanacearum* both in vitro and in vivo, as well as on disease severity. We found that indirect effects provided far better prediction of *R. solanacearum* density in vitro (Fig. 5A and Table 1) and in vivo (Fig. 5B and Table 1) and bacterial wilt disease severity (Fig. 5C and Table 1), as compared to direct effects on the pathogen. Together, these results suggest that indirect effects of rhizobacteria on the helpers’ growth predicted pathogen density better than direct effects on the pathogen itself.

## Discussion

In this study, we evaluated the prevalence of pathogen-helper bacterial strains in the tomato rhizosphere microbiome as well as the potential to target such helpers for microbiome management strategies aiming to reduce pathogen growth. As a model pathogen, we used *Ralstonia solanacearum*, a widespread and problematic phytopathogenic bacterium that causes wilt diseases on tomatoes and more than 200 economically important crops and ornamentals [30]. Combining in vitro and in vivo approaches, we compared the influence of the direct (i.e., on *R. solanacearum* growth directly) vs. the indirect (i.e. on the growth of *R. solanacearum* helper strains) effects of tomato-associated rhizobacteria on the growth of the pathogen as well as subsequent development of disease symptoms. Overall, indirect effects, i.e. inhibition of helper strains, were the major determinants of pathogen suppression as compared to direct impacts on the pathogen itself. To our knowledge, this represents the first demonstration of such an indirect strategy for the potential suppression of soil-borne plant disease.

The isolated rhizobacteria in this study belonged to four major phyla (*Proteobacteria, Firmicutes, Bacteroidetes* and *Actinobacteria*), which are collectively presumed to be copiotrophs [31, 32] and are known to be dominant phyla found in the rhizosphere [33]. We found facilitation to be widespread, with half of the tested isolates (50.6%) promoting pathogen growth using a supernatant assay. This result adds to the recent insights that many microorganisms may act as helpers of pathogens in the rhizosphere [11]. Indeed, different bacterial strains affiliated with *Bacillus* and *Microbacterium* were previously shown to have a modest but significant stimulatory effect on the growth of *R. solanacearum* and a promotion of disease development [11]. Also, some fungi affiliated with *Ascomycetes, Basidiomycetes* and *Zygomycetes* have been shown to facilitate pathogen entry into tomato roots by producing chlamydospores that can host *R. solanacearum* cells [34]. Moreover, one *Pseudomonas* phylotype was found to exacerbate disease symptoms in tomato plants by establishing commensal interactions with an oomycete pathogen to increase its access to plant nutrients [35]. Interestingly, most of the helpers in our study belonged to the *Proteobacteria* (49.1%) and *Actinobacteria* (63.9%), two phylogenetic groups that are often highlighted for containing bacteria that are effective at suppressing pathogen growth [36, 37]. Our results thus call for a rethinking of the interactions that shape the microbiome, with the realization that facilitation is widespread and important. However, it should be noted that the rhizobacterial collection we utilized in this study clearly does not provide a full taxonomic inventory of the rhizosphere microbiome. For instance, the medium we used most likely selected for more copiotrophic strains from the full soil microbiome. Such copiotrophs might have different effects on pathogen growth as compared to more oligotrophic bacteria, because they typically have higher growth rates and lower substrate affinities [38].

Several mechanisms have been put forth to explain mutualism or commensalism among bacteria, mainly as related to the benefits gained from the use of metabolites processed by another member of the community [39]. For instance, peptidoglycan produced by *Bacillus cereus* may promote the growth of several bacterial strains affiliated with the *Cytophaga-Flavobacterium* group [40]. Siderophores produced by microorganisms can also be accepted as public goods by other bacteria with siderophore protein receptors to obtain limited iron in the environment to maintain growth and metabolism, hence increasing population biomass [41]. Although beyond the scope of the current study, the promoting mechanisms of the helper strains towards *R. solanacearum* are most likely related to certain metabolites, as promotion was also observed using supernatant assays (Figs. 2A and 3A).

In general, the indirect effects of the rhizobacteria we studied were the best predictors of *R. solanacearum* density and the realized level of plant disease severity (Fig. 4D, E, F). It is noteworthy that the level of variation explained by indirect effects was higher in our in vitro assays (75% and 79%, Fig. 4D) as compared to our in vivo experiments (less than 50%, Fig. 4E, F). This difference might be explained by the more open nature of the plant rhizosphere as compared to the in vitro setting. Variation in environmental aspects such as soil structure and the resident microbiome also could contribute to a great level of variation in realized pathogen density. Alternatively, microbial impacts on plant immunity might also impact the level of plant disease observed [42, 43]. For instance, several bacterial secondary metabolites involved in pathogen suppression may also impact plant immunity: for example, 2,4-diacetylphloroglucinol (DAPG) produced by fluorescent *Pseudomonas* spp. [44] or lipopeptide surfactins produced by *Bacillus subtilis* [45] have such a dual function.

The rhizobacterial strains used in this study exhibited a wide range of effects on the pathogen and its bacterial helper strains. Many of them inhibited both *R. solanacearum* as well as its helpers. Our model on the interaction scenarios where rhizobacterial strains inhibited both the pathogen and its helpers (quadrant “H^−^P^−^” in Fig. 3C) showed that inhibition of the helper strains was a more effective path toward *R. solanacearum* reduction than direct inhibition effects on the pathogen itself. Even if a biocontrol agent is active against *R. solanacearum* [4, 18], its efficiency in reality may be more due to its interaction with indigenous helpers. We therefore propose that strategies for integrated biological control of the pathogen need to be reconsidered to incorporate indirect effects on pathogen helpers to provide more ecological solutions to combat soil-borne pathogens. Although the underlying mechanisms of helper inhibition still need to be unraveled and our communities here were far less diverse and far simpler than natural communities, our findings contribute to our knowledge of rhizobacteria-pathogen interactions and provide a new potential strategy for efficient and sustainable biological control of soil-borne pathogens.

## Supplementary information

Supplementary Figures and Tables

Model

Library of rhizobacterial strains
