# Supplementary material for: Indirect reduction of Ralstonia solanacearum via pathogen helper inhibition
Source: ISME J. 2021 Oct 20;16(3):868–75. doi: 10.1038/s41396-021-01126-2 (PMC8857195; doi:10.1038/s41396-021-01126-2)
Supplement: Supplementary file 1 — Supplementary Figures and Tables [file 41396_2021_1126_MOESM1_ESM.pdf]

Supplementary Information for

Indirect reduction of *Ralstonia solanacearum* via pathogen helper inhibition

Mei Li[1,6], Thomas Pommier[2], Yue Yin[1], Jianing Wang[1], Shaohua Gu[3], Alexandre Jousset[1], Joost Keuskamp[4,6], Honggui Wang[5], Zhong Wei[1, *], Yangchun Xu[1], Qirong Shen[1] and George A. Kowalchuk[6]

# Corresponding author: Zhong Wei, Email: weizhong@njau.edu.cn

Materials and methods

Measuring the direct effects of rhizobacteria on pathogen growth by co-culture assay *in vitro*

We used *Ralstonia solanacearum* strain QL-Rs1115 tagged with the pYC12-mCherry plasmid as an invading bacterial pathogen[1, 2]. We first tested the effects of 515 bacterial strains on the growth of *R. solanacearum in vitro* by using co-culture assays. All strains were first grown alone in liquid NA medium (glucose 10.0 g l^{-1}, tryptone 5.0 g l^{-1}, yeast extract 0.5 g l^{-1}, beef extract 3.0 g l^{-1}, pH 7.0) on a shaker at 170 rpm, 30°C overnight. All rhizobacterial strain densities were adjusted to ~10^7 cells per ml and the density of mCherry fluorescence-tagged *R. solanacearum* QL-Rs1115-RFP to ~10^6 cells per ml. Co-cultures with even starting volumes (50%:50%) were set up in 96-well plates with liquid NA medium. Each treatment was replicated three times. All bacterial cultures were grown for 48 h at 30°C with shaking (170 rpm) before measuring pathogen density as red mCherry protein fluorescence intensity (excitation: 587 nm, emission: 610 nm) using a SpectraMax M5 plate reader [3, 4]. To test for significance of growth promotion or inhibition, *R. solanacearum* densities (RFP) were log10-transformed prior to analyses of variance (ANOVA) and Bonferroni t-test to compare mean differences between each rhizobacterial supernatant treatments and the control treatment, with p-values below 0.05 were considered statistically significant. The effect on pathogen growth was defined as the percentage of improvement or reduction in pathogen growth by the supernatant

compared with the control treatment. When the effect on pathogen growth was positive, *i.e.* when the supernatants from strains significantly promoted the growth of the pathogen, they were considered as helpers of the pathogen. If the effect on pathogen growth was negative, *i.e.* when the supernatants from strains significantly inhibited the growth of the pathogen, they were considered as inhibitors of the pathogen.

Measuring the relationship between fluorescence units and CFU

To link the fluorescence units and CFU of *R. solanacearum*, we measured the relationship between them. Briefly, we grew 2 μl of the *R. solanacearum* strain, which was tagged with the pYC12-mCherry plasmid, in 96-well plates with 198 μl NB (nutrient broth) medium (glucose 10.0 g l-1, tryptone 5.0 g l-1, yeast extract 0.5 g l-1, beef extract 3.0 g l-1, pH 7.0) in each well on a shaker at 170 rpm, 30°C. Pathogen growth was measured as fluorescence units (excitation: 587 nm, emission: 610 nm) using a SpectraMax M5 plate reader, as well as colony number units (CFU) by serial dilution per 200 μl. This allowed us to assess the relationship between these two measures.

Table S1. Detailed information of 12 rhizobacterial strains used in *in vivo* experiments with tomato plant.

Rhizobacteria ID	Effect on pathogen growth	Effect on Pi growth	Effect on Mp growth	Taxonomy annotation (Phylum_Class_Order_Family_Genus)
45	-0.85806	-0.27201	-0.53868	Firmicutes_Bacilli_Bacillales_Bacillaceae_Bacillus
143	-0.35453	-0.41969	0.278354	Bacteroidetes_Bacteroidia_Flavobacteriales_Weeksellaceae_Chryseobacterium
183	-0.58988	-0.63446	0.257473	Firmicutes_Bacilli_Bacillales_Bacillaceae_Lysinibacillus
199	-0.49369	-0.30745	-0.67538	Firmicutes_Bacilli_Bacillales_Bacillaceae_Bacillus
277	-0.91819	-0.69634	-0.71173	Proteobacteria_Gammaproteobacteria_Enterobacteriales_Enterobacteriaceae_Pantoea
322	-0.18957	-0.28565	-0.4469	Bacteroidetes_Bacteroidia_Flavobacteriales_Weeksellaceae_Chryseobacterium
372	-0.01369	-0.69241	-0.85189	Firmicutes_Bacilli_Bacillales_Staphylococcaceae_Staphylococcus
433	-0.40062	-0.56357	-0.60598	Firmicutes_Bacilli_Bacillales_Bacillaceae_Bacillus
477	-0.57756	-0.48129	-0.65465	Firmicutes_Bacilli_Bacillales_Planococcaceae_Lysinibacillus
525	-0.94817	-0.60577	-0.77783	Proteobacteria_Gammaproteobacteria_Enterobacteriales_Enterobacteriaceae_Salmonella
549	-0.87933	-0.62954	-0.75755	Proteobacteria_Gammaproteobacteria_Enterobacteriales_Enterobacteriaceae_Enterobacter
563	-0.94013	-0.69662	-0.80412	Proteobacteria_Gammaproteobacteria_Enterobacteriales_Enterobacteriaceae_Pantoea

Table S2. Treatments used in *in vivo* experiments with tomato plants. Table rows show different treatments and table columns show the absence (0) or presence (1) of given species in these treatments. The last column shows which rhizobacteria (see Table S1 the details) we used in each treatment.

Treatments	Pathogen_Rs	Helper_Pi	Helper_Mp	Rhizobacteria ID
1	0	0	0	0
2	1	0	0	0
3	1	1	0	0
4	1	0	1	0
5	1	1	0	45
6	1	1	0	143
7	1	1	0	183
8	1	1	0	199
9	1	1	0	277
10	1	1	0	322
11	1	1	0	372
12	1	1	0	433
13	1	1	0	477
14	1	1	0	525
15	1	1	0	549
16	1	1	0	563
17	1	0	1	45
18	1	0	1	143
19	1	0	1	183
20	1	0	1	199
21	1	0	1	277
22	1	0	1	322
23	1	0	1	372
24	1	0	1	433
25	1	0	1	477
26	1	0	1	525
27	1	0	1	549
28	1	0	1	563

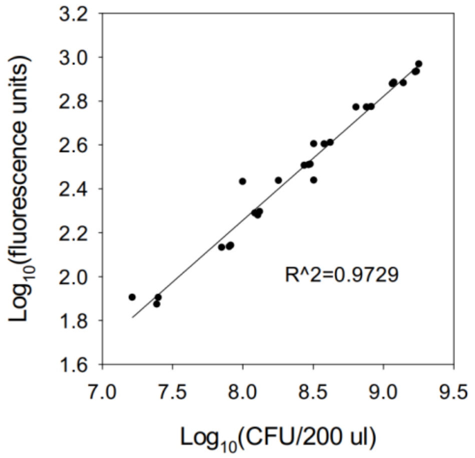

Fig. S1. The relationship between mCherry fluorescence intensity and CFU of *Ralstonia solanacearum.*

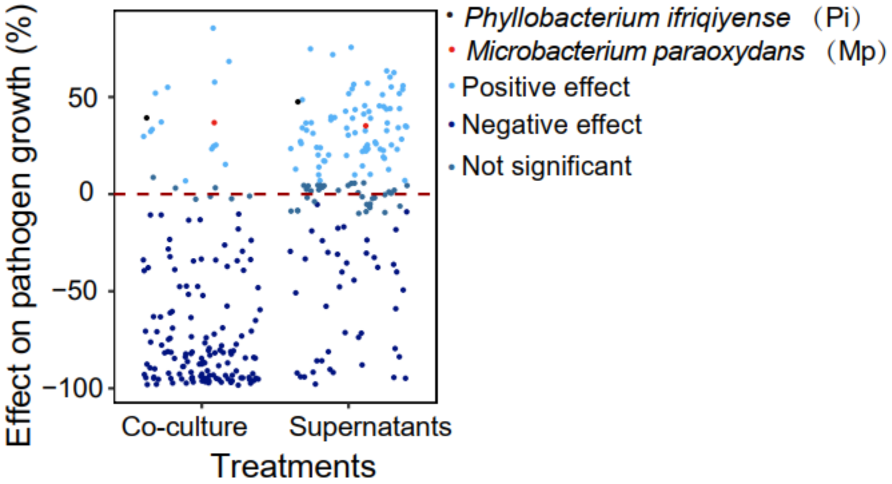

Fig. S2. Effects of 160 rhizobacterial strains on *Ralstonia solanacearum* growth in both co-culture and supernatant assays. *Phyllobacterium ifriqiyense* (Pi) and *Microbacterium paraoxydans* (Mp) were considered helper strains which showed strong positive effects on *R. solanacearum* growth both in co-culture and supernatant assays. Each dot represents one rhizobacteria in the figure.

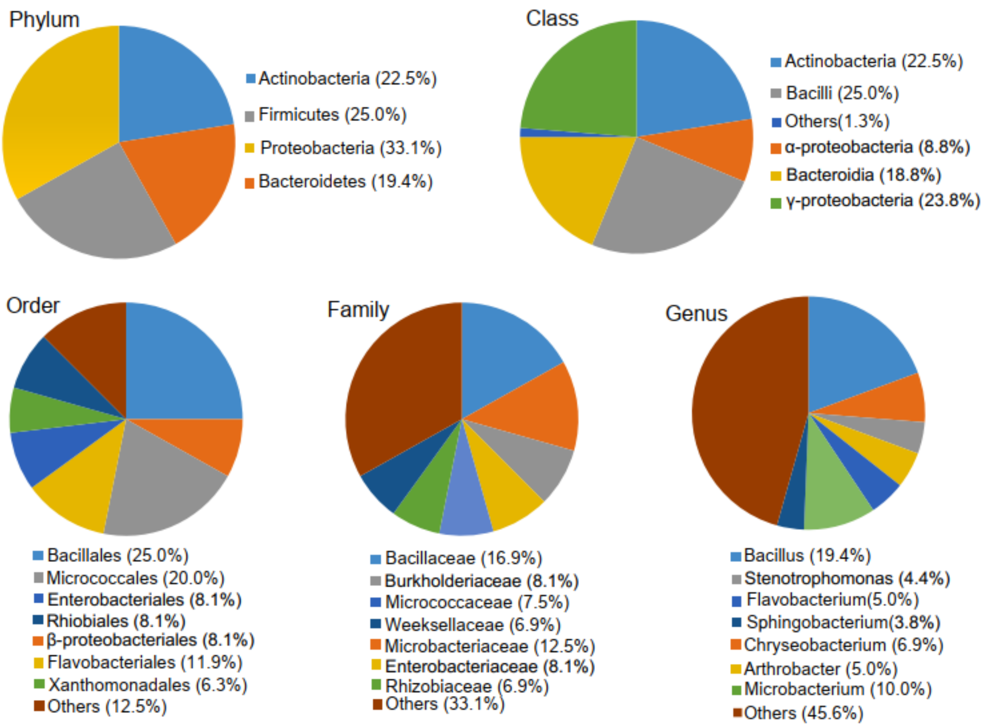

Fig. S3. The diversity and taxonomic classification of rhizosphere bacterial isolates. A total of 160 rhizosphere isolates were identified by 16s rRNA sequencing and their closest relatives were determined using the NCBI database. Seven bacterial groups with highest relative abundances at the phylum, class, order, family, and genus levels are shown in the figure, while groups with relatively low abundances were merged and are presented as one group 'Others'. In all panels, percentage (%) values in brackets represent the proportion of each bacterial group of the total isolates (160 bacterial isolates).

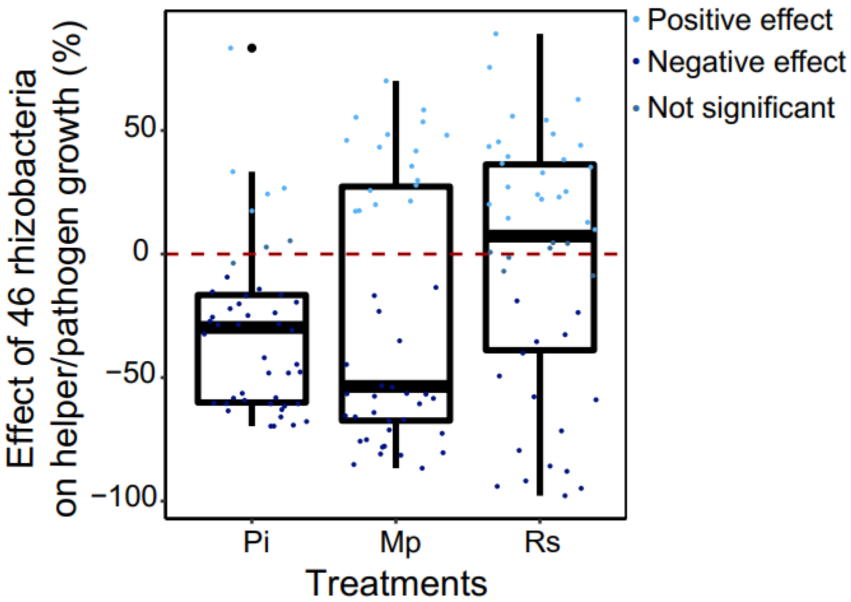

Fig. S4. Effects of 46 rhizobacteria from 160 on helper (*Phyllobacterium ifriqiyense* Pi or *Microbacterium paraoxydans* Mp) or *Ralstonia solanacearum* (Rs) growth in supernatant assay. A subset of 46 rhizobacterial strains covering a gradient of positive, neutral or negative interaction with the pathogen based on supernatant assays was chosen, which also varying in their effect on two helpers. Each dot represents one rhizobacteria in the figure.

References

1. Wei, Z., et al., *Efficacy of Bacillus-fortified organic fertiliser in controlling bacterial wilt of tomato in the field.* Applied Soil Ecology, 2011. **48**(2): p. 152-159.
2. Wei, Z., et al., *Trophic network architecture of root-associated bacterial communities determines pathogen invasion and plant health.* Nat Commun, 2015. **6**: p. 8413.
3. Gu, S., et al., *Competition for iron drives phytopathogen control by natural rhizosphere microbiomes.* Nat Microbiol, 2020.
4. Li, M., et al., *Facilitation promotes invasions in plant-associated microbial communities.* Ecol Lett, 2019. **22**(1): p. 149-158.
